# AKT2 Blocks Nucleus Translocation of Apoptosis-Inducing Factor (AIF) and Endonuclease G (EndoG) While Promoting Caspase Activation during Cardiac Ischemia

**DOI:** 10.3390/ijms18030565

**Published:** 2017-03-06

**Authors:** Shuai Yang, Xinmei Zhao, Hui Xu, Fan Chen, Yitao Xu, Zhe Li, Daniel Sanchis, Liang Jin, Yubin Zhang, Junmei Ye

**Affiliations:** 1 State Key Laboratory of Natural Medicines, Department of Biochemistry, School of Life Science and Technology, China Pharmaceutical University, Nanjing 210006, China; yangshuai923@163.com (S.Y.); 14211030585@cpu.edu.cn (X.Z.); fancy0703@163.com (F.C.); yito-xu@outlook.com (Y.X.); liangjin1975@cpu.edu.cn (L.J.); 2 Center of Translational Medicine and Jiangsu Key Laboratory of Molecular Medicine, Medical School of Nanjing University, Nanjing 210093, China; hxu@diacarta.com; 3 Department of Cardiology, Renmin Hospital of Wuhan University, Wuhan 430060, China; zhelizl@gmail.com; 4 Cardiovascular Research Institute, Wuhan University, Wuhan 430060, China; 5 Hubei Key Laboratory of Cardiology, Wuhan 430060, China; 6 Institut de Recerca Biomèdica de Lleida (IRBLLEIDA)—Universitat de Lleida, Edifici Biomedicina-I. Av. Rovira Roure, 80., 25198 Lleida, Spain; daniel.sanchis@cmb.udl.cat

**Keywords:** cardiomyocytes, apoptosis, protein kinase B2 (AKT2), ischemia, apoptosis-inducing factor (AIF), endonuclease G (EndoG)

## Abstract

The AKT (protein kinase B, PKB) family has been shown to participate in diverse cellular processes, including apoptosis. Previous studies demonstrated that protein kinase B2 (AKT2^−/−^) mice heart was sensitized to apoptosis in response to ischemic injury. However, little is known about the mechanism and apoptotic signaling pathway. Here, we show that AKT2 inhibition does not affect the development of cardiomyocytes but increases cell death during cardiomyocyte ischemia. Caspase-dependent apoptosis of both the extrinsic and intrinsic pathway was inactivated in cardiomyocytes with AKT2 inhibition during ischemia, while significant mitochondrial disruption was observed as well as intracytosolic translocation of cytochrome C (Cyto C) together with apoptosis-inducing factor (AIF) and endonuclease G (EndoG), both of which are proven to conduct DNA degradation in a range of cell death stimuli. Therefore, mitochondria-dependent cell death was investigated and the results suggested that AIF and EndoG nucleus translocation causes cardiomyocyte DNA degradation during ischemia when AKT2 is blocked. These data are the first to show a previous unrecognized function and mechanism of AKT2 in regulating cardiomyocyte survival during ischemia by inducing a unique mitochondrial-dependent DNA degradation pathway when it is inhibited.

## 1. Introduction

Apoptosis is defined as programmed cell death (PCD), which is executed by a family of cysteinyl aspartate proteinases known as caspases [1]. Caspases belong to a family of highly conserved aspartate-specific cysteine proteases that are expressed as inactive zymogens in most animal cells and serve as the central executioners of apoptosis [2]. When activated, these enzymes cleave their protein substrates on the carboxy-terminal side of an aspartate residue [3]. There are two signaling pathways involved in PCD. The death receptor pathway (the extrinsic pathway), involved in the activation of caspase-8 and -10, is initiated when death receptors are stimulated. An alternative pathway (the intrinsic pathway) involves the activation of caspase-9, which relies on the release of cytochrome c (Cyto C) and other mitochondrial proteins in the cytoplasm, and this process is controlled by B-cell lymphoma-2 (Bcl-2) family proteins [4]. Once caspases-3, -6, and -7 are activated, they cleave a variety of proteins or death substrates. During early apoptosis, caspase-3 cleaves poly (ADP-ribose) polymerase (PARP), which decreases its DNA repair activity and leads to apoptotic cell death [5].

Mitochondria play a key part in the regulation of apoptosis [6]. Endonuclease G (EndoG), a well-conserved nuclease, is a homodimer that is considered to be involved in mitochondrial DNA replication with important roles in DNA recombination and repair. EndoG is localized in the mitochondria, and then translocates to the nucleus during apoptosis with cell death stimuli such as truncated BH3 interacting-domain death agonist (tBid), tumor-necrosis factor-alpha (TNF-α) and ultraviolet (UV) irradiation [7]. Biochemical and genetic studies have identified EndoG as one of the endonucleases important for mammalian DNA fragmentation during apoptosis [7]. Apoptosis-inducing factor (AIF) is normally confined to mitochondria but translocates to the nucleus in response to specific death signals [8,9]. It is reported that AIF causes high molecular weight DNA fragmentation and chromatin condensation in cells and isolated nuclei in a caspase-independent manner [9,10]. Previous research by Bahi and coworkers has demonstrated that the release of EndoG together with AIF from mitochondria to cytosol in rat postnatal differentiated cardiomyocytes is induced by experimental ischemia and causes DNA degradation in cardiomyocytes thereafter [11]. However, the involvement of EndoG and AIF in cardiomyocytes with AKT2 inhibition during ischemia still remains unclear.

AKT is a serine/threonine protein kinase that is activated by a number of growth factors or cytokines in a phosphatidylinositol-3 kinase (PI3K)-dependent manner [12]. The AKT family consists of three isoforms—AKT1, AKT2 and AKT3, each of them encoded by distinct, highly conserved genes [13]. All three isoforms are expressed in the myocardium with AKT1 and AKT2 comprising the vast majority of total AKT protein in the heart [14]. Of the three isoforms of AKT, AKT2 is reported to play a critical role in antagonizing cardiomyocyte apoptosis that occurs in response to a variety of stimuli [15]. However, the function of AKT2 deficiency on cardiac ischemia/reperfusion (I/R) injury is controversial, with most reports suggesting AKT2^−/−^ cardiomyocytes were sensitized to apoptosis [13,15,16], and one supporting a beneficial effect during cardiac I/R [17]. Despite the controversy of AKT2 function on cardiac I/R injury, the molecular mechanisms regulating this cell death remain poorly defined.

To gain a better understanding of the AKT2 isoform in the heart during ischemia, we used specific AKT2 inhibitor to directly examine the role of AKT2 during ischemia-induced apoptosis and the signaling pathway in neonatal rat cardiomyocytes (NRCMs) because of the low survival of primary cardiomyocytes directly obtained from the heart of AKT2 knockout (KO) mice for in vitro study. In our study, we demonstrate that ischemic stimuli induce the release of mitochondrial factors involved in cell death induction in NRCMs with AKT2 inhibition. Indeed, we show that the mitochondrial outer membrane permeabilization induced by bcl-2-like protein 4 (Bax) results in the release of apoptotic factors including Cyto C, AIF and EndoG into the cytosol and causes cell death by inducing DNA degradation without caspase activation when AKT2 is blocked. Our results provide the evidence that AIF and EndoG are two important factors involved in the regulation of caspase-independent cell death induced by ischemia-mediated cardiomyocte injury when AKT2 is inhibited.

Our study provides a novel view of AKT2 function on ischemic NRCMs that was not elucidated previously and hence gains a better understanding of different cell death pathways that are provoked in ischemic NRCMs with AKT2 inhibition.

## 2. Results

### 2.1. AKT2 Inhibition Induces Elevation of Apoptotic Gene Expression in Myocardium without Affecting Myocyte Development

**Figure 1:**
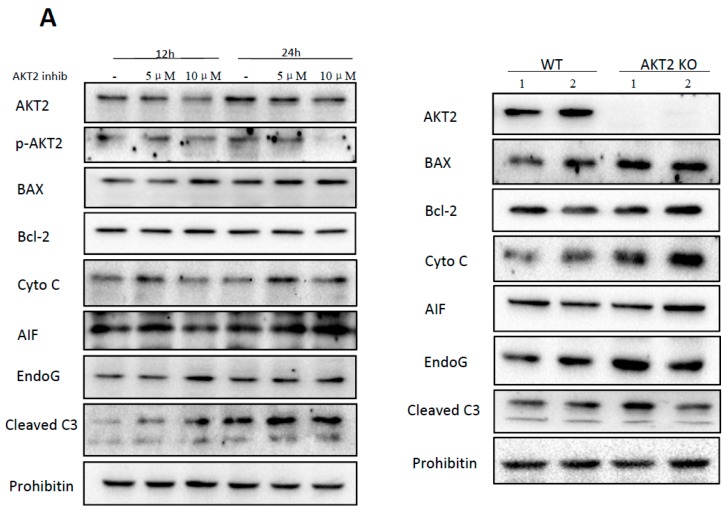

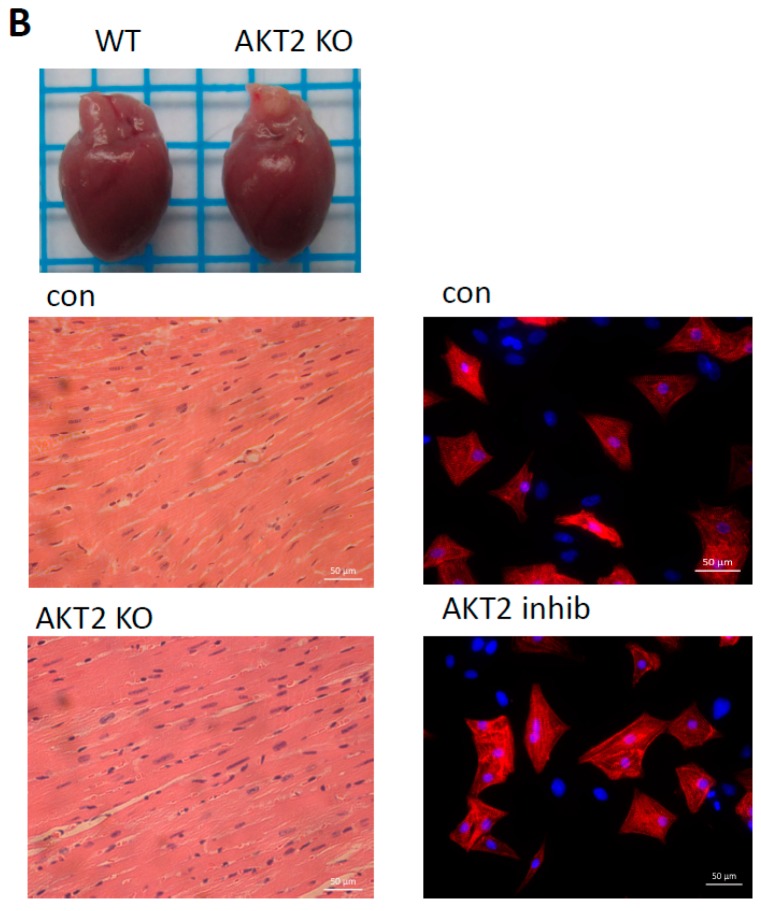
AKT2 inhibition triggers apoptosis-related gene expression without affecting cardiac cell morphology. (**A**) left panel: expression of AKT2, phosphorylated AKT2 (p-AKT2), Bax, Bcl-2, cytochrome C (Cyto C), apoptosis-inducing factor (AIF), endonuclease G (EndoG) and cleaved caspase-3 (cleaved C3) proteins was detected in neonatal cardiomyocytes from 3-day-old rat pups treated with or without AKT2 inhibitor at the concentration of 5 and 10 μM for 12 and 24 h respectively. Right panel: expression of proteins the same as (**A**) detected in wild type (WT) and AKT2 knockout (KO) mice hearts at 4 months old, 1 and 2 refers to different numbers of heart sample; (**B**) left panel: hearts from WT or AKT2 KO mice at 4 months old and HE staining (×400). Right panel: Immunofluorescence staining of α-actinin (**red**) and hoechst (**blue**) in neonatal rat cardiomyocytes (NRCMs) cultured with or without 10 μM AKT2 inhibitor (AKT2 inhib) for 12 h (×400). Data are repeated for three independent experiments.

In order to investigate the role of AKT2 in the myocyte during ischemia, we initially compared apoptosis-related genes and observed slight increase of genes involved in mitochondrial-dependent apoptosis in NRCMs treated with a specific AKT2 inhibitor (Figure 1A, left panel). The same result was also observed in AKT2 knockout (AKT2 KO) mice hearts when compared with wild-type (WT) hearts (Figure 1A, right panel). To ascertain if NRCM development is affected by the changes of apoptotic gene expression, we compared hearts of AKT2 KO and WT mice at the age of four months; there was little difference in either of the heart weights (Figure 1B, left panel). WT and AKT2 KO hearts with hematoxylin-eosin (HE) staining indicated that these myocytes have well-ordered myofibrils with a distinct sarcomeric registry (Figure 1B, left panel), which is consistent with a previous study [18]. Moreover, structures of cardiomyocytes treated with or without AKT2 inhibitors were observed by immunofluorescence, which showed no difference in cell morphology and area when compared with control (Figure 1B, right panel), suggesting no significant morphological changes in cardiomyocyte development due to AKT2 ablation both in vivo and in vitro. Our results indicate that the absence of AKT2 per se is a trigger of mitochondrial apoptotic genes in the heart but does not influence cardiomyocyte development under normal circumstance.

### 2.2. Blockage of AKT2 Facilitates Apoptosis in Cardiomyocytes

**Figure 2:**
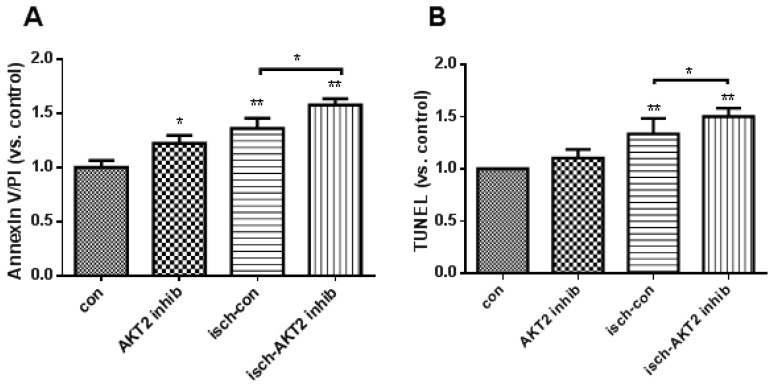

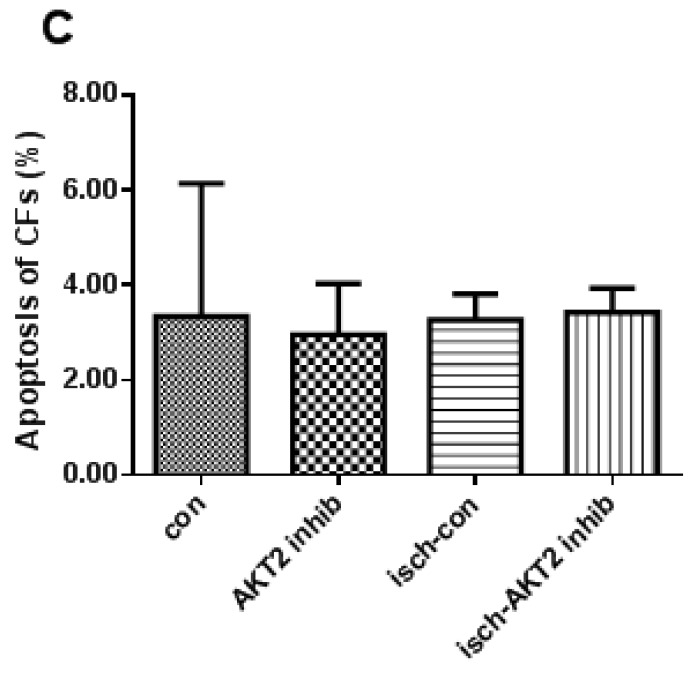
Increased apoptosis in cardiomyocytes treated with AKT2 inhibitor during ischemia. (**A**) Detection of Annexin V/Propidium iodide (PI) in cardiomyocytes treated with or without AKT2 inhibitor (AKT2-inhib, 10 μM) and cultured under normal (con) or ischemia (isch) conditions for 12 h by flow cytometry; (**B**) TUNEL (transferase-mediated deoxyuridine triphosphate-biotin nick end labeling) assay of NRCMs with the same treatment as (**A**); (**C**) Quantification of apoptotic cardiomyocyte fibroblasts (CFs) treated with or without AKT2 inhibitor during ischemia by flow cytometry. Values are percentage of apoptotic cells versus total cells in plate. The bars represent the mean ± SEM (standard error of mean) of three independent experiments. * *p* < 0.05, ** *p* < 0.01 vs. control (one-way ANOVA followed by a Tukey test for post hoc analyses).

In order to explore the role of AKT2 in cardiac apoptosis during ischemia, cultured NRCMs were treated with AKT2 inhibitor for 3 h before hypoxia treatment, afterwards experimental ischemia (hereafter, ischemia) was achieved by culturing cells inside a hypoxic incubator in Tyrode’s solution without serum and glucose. Increased apoptosis was observed in AKT2 inhibitor-treated cardiomyocytes compared with control during ischemia (Figure 2A,B). Since cardiac fibroblasts (CFs) are resistant to apoptosis due to their strong expression of the anti-apoptotic factor Bcl-2 [19], in order to exclude the potential apoptosis stimulated in CFs, which usually exists as contamination during primary cardiomyocyte isolation, apoptosis was also checked in CFs treated with AKT2 inhibitor. The results showed that the portion of apoptotic CFs maintained almost the same level of resistance to apoptosis as control when AKT2 is blocked or not during ischemia (Figure 2C). These data infer that AKT2 is responsible for cardiac apoptosis under ischemic stimuli and establishes a correlation between AKT2 inhibition and the elevated apoptosis during cardiac hypoxia.

### 2.3. AKT2 Blockage Diminishes the Activation of Extrinsic Apoptotic Signaling Pathway

**Figure 3:**
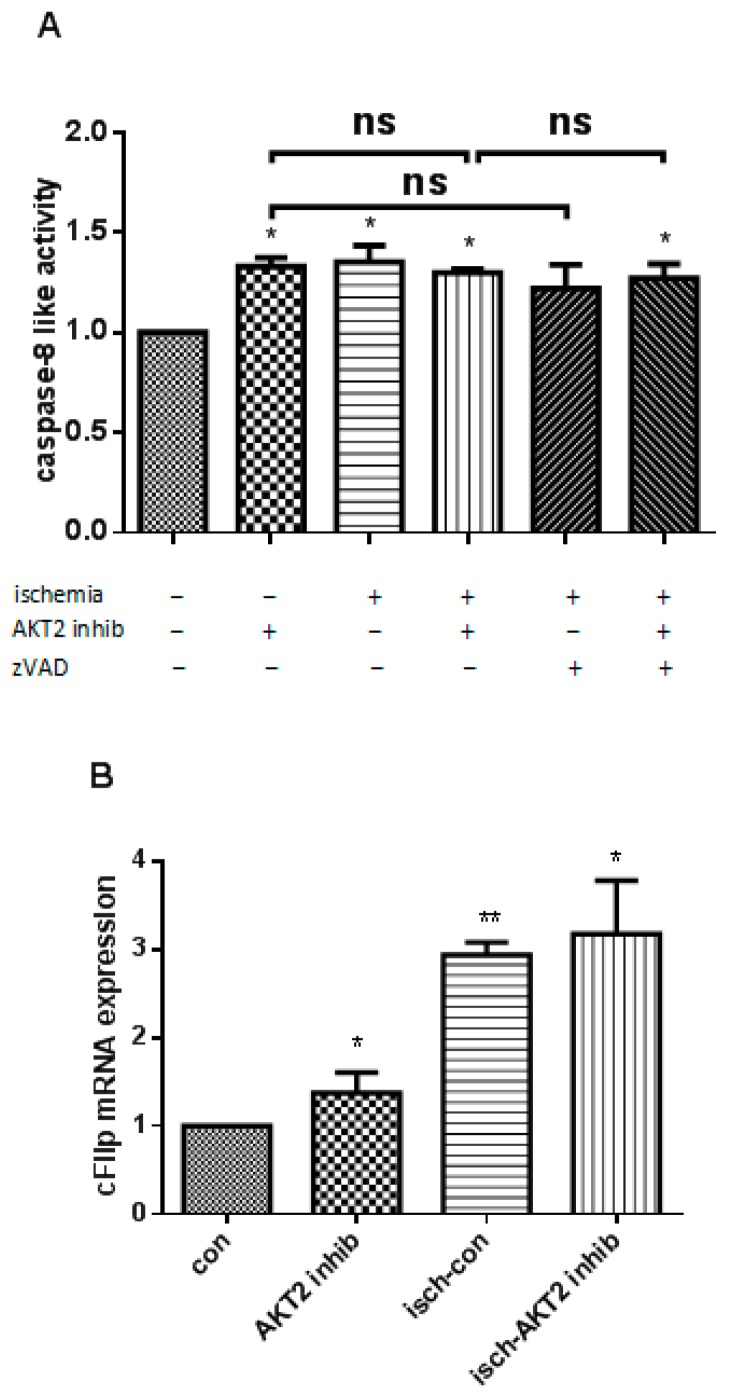
Inactivation of caspase-dependent apoptosis in ischemic cardiomyocytes treated with an AKT2 inhibitor. (**A**) Initiator caspase-8 activity in extracts of cardiomyocytes with con, an AKT2 inhibitor, and ischemic cardiomyocytes treated with or without AKT2 inhibitor and/or zVAD; (**B**) Quantitative real time PCR of cFlip transcript in cardiomyocytes with the same treatment as in A. zVAD: pan-caspase inhibitor z-VAD-fmk, 100 μM. The bars represent the mean ± SEM of three independent experiments. * *p* < 0.05, ** *p* < 0.01 vs. control (one-way ANOVA followed by a Tukey test for post hoc analyses). ns: not significant.

We further aimed at providing functional evidence for the role of death receptor-dependent (extrinsic) apoptotic pathway in the promotion of ischemic cardiomyocytes with AKT2 inhibition. Initiator caspase-8-like activity was measured as an indication of the provocation of the death receptor-dependent pathway during ischemic cell death. Although ischemic cardiomyocytes treated with AKT2 inhibitor maintained the same level of executioner caspase-8 activity as ischemic NRCMs which sustained significantly higher activated caspase-8 than control, there was no significant difference when compared with cardiomyocytes treated with AKT2 inhibitors cultivated under normal conditions (Figure 3A). This did not show increased apoptosis as proven in Figure 2B. To verify this result, pan-caspase inhibitor zVAD-fmk was added to cardiomyocytes during ischemia and caspase-8 activity from cell extracts was evaluated. Our data shows no obvious change of caspase-8 activity among different treatments (Figure 3A). Moreover, we further ascertained an increased expression at transcriptional level for caspase FLICE-like inhibitory protein (cFlip), a natural inhibitor of caspase-8 activity [20], upon ischemia induction (Figure 3B). These data support the conclusion that upon AKT2 inhibition, ischemia is not a further stimulus for the activation of the extrinsic apoptotic pathway in cardiomyocytes.

### 2.4. AKT2 Inhibition Promotes Mitochondrial Membrane Injury Independent of Intrinsic Apoptotic Pathway

**Figure 4:**
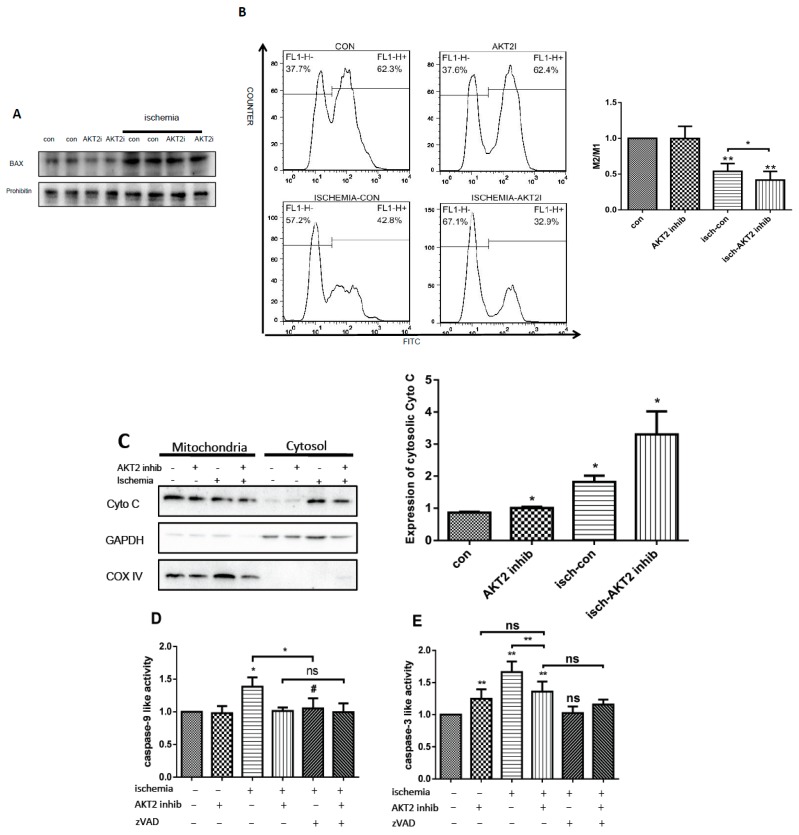

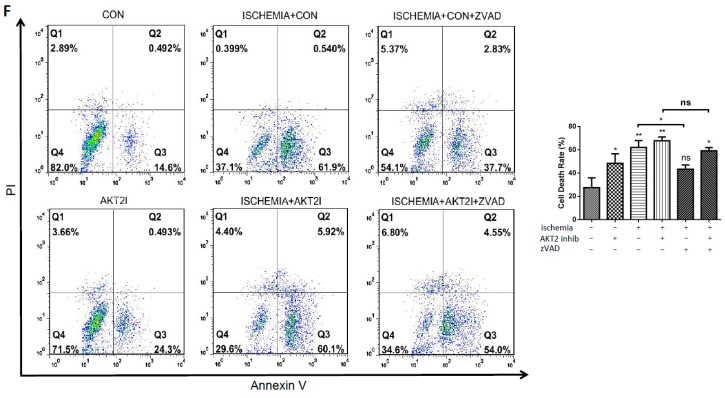
AKT2 inhibition promotes mitochondrial injury and cell death without intrinsic apoptosis activation during cardiac ischemia. (**A**) Upper panel: Bax abundance was analyzed in total protein extracts of cardiomyocytes with different treatment; lower panel: Quantification of Bax protein abundance from western blot. akt2i: AKT2 inhibitor; (**B**) Mitochondrial outer membrane potential (MOMP) was evaluated by flow cytometry in normal and ischemic cardiomyocytes treated with or without an AKT2 inhibitor; (**C**) Left panel: Cytochrome C (Cyto C) in mitochondrial and cytosolic extracts in cardiomyocytes treated with or without an AKT2 inhibitor under normal conditions or ischemia; right panel: Densitometry of western blot bands which was performed with Image J software; (**D**,**E**) Executioner caspase-9 and -3 activity measured in extracts of cardiomyocytes with different treatment; (**F**) Detection of AnnexinV/PI by flow cytometry in cardiomyocytes with different treatment as shown in the figure. zVAD, pan-caspase inhibitor z-VAD-fmk, 100 μM; The bars represent the mean ± SEM of three independent experiments. * *p* < 0.05, ** *p* < 0.01 vs. control; # *p* < 0.05 vs. cells with ischemia treatment; ns: not significant vs. con (one-way ANOVA followed by a Tukey test for post hoc analyses).

To further investigate the mechanism involved in cardiac apoptosis during ischemia when AKT2 is inhibited, the role of the caspase-dependent intrinsic apoptotic pathway was evaluated. AKT2 is proven to be an inhibitor of Bax [21,22], the induction of which results in downstream programming of mitochondrial dysfunction as well as activation of caspase [23,24]. Ischemic cardiomyocytes with AKT2 inhibition maintain much higher Bax protein abundance (Figure 4A), indicating the provocation of mitochondrial-dependent apoptosis. Mitochondrial-dependent apoptosis happens following the disruption of mitochondrial membranes and the release of apoptotic proteins located in the intermembrane space thereafter. First to evaluate mitochondrial integrity, mitochondrial outer membrane potential (MOMP) was assessed and the result indicates serious mitochondrial disruption (Figure 4B), suggesting increased mitochondrial permeability and the possible release of mitochondrial-located proteins.

Since the cytosolic translocation of Cyto C is deemed as the main inducer of mitochondiral-dependent apoptosis [25,26,27], the cytosolic distribution of Cyto C was investigated in cardiomyocytes with different treatment and the result shows much more intracytosolic translocation of Cyto C in ischemic cardiomyocytes with AKT2 blockage (Figure 4C). However, activated caspase-9, an initiator caspase which induces apoptosis upon Cyto C translocation to cytosol, remained at normal levels in ischemic cardiomyocytes treated with an AKT2 inhibitor, compared with ischemic (isch–con)cardiomyocytes (Figure 4D), which obtained significant increase. Moreover, caspase-3 activity was also evaluated and the result suggested equivalent level of caspase-3 activity in ischemic NRCMs treated with AKT2 inhibitor compared with cardiomyocytes with AKT2 inhibition under normal conditions, although there were significant changes compared with control (Figure 4E). These results indicate that caspase-dependent intrinsic apoptosis is not activated, although mitochondrial injury occurs during myocardium ischemia when AKT2 is inhibited. Our results also suggested the participation of caspase-dependent apoptosis during cardiomyocyte ischemia without AKT2 inhibition. To further verify this conclusion, cardiomyocytes were treated with pan-caspase inhibitor zVAD in addition to AKT2 inhibitor during ischemia, and cell apoptosis as well as caspase-9 and -3 activity was evaluated (Figure 4D–F). The data suggested apoptosis was partly blocked in zVAD-treated NRCMs during ischemia, while there was no change in cardiomyocytes with either AKT2 inhibition or zVAD-treatment. The decrease of caspase-9 and -3 activity was significant between cardiomyocytes treated with and without zVAD during ischemia, while there were no obvious changes when AKT2 was inhibited. Taken together, our results above indicate that AKT2 inhibition induces the silencing of caspase-dependent apoptosis of the internal signaling pathway in cardiomyocytes during ischemia and hence prompts a distinct cell death signaling pathway that could be provoked by AKT2 inhibition.

### 2.5. Cardiac AKT2 Inhibition Facilitates EndoG and AIF Cytosolic and Nucleus Translocation from Mitochondria

**Figure 5:**
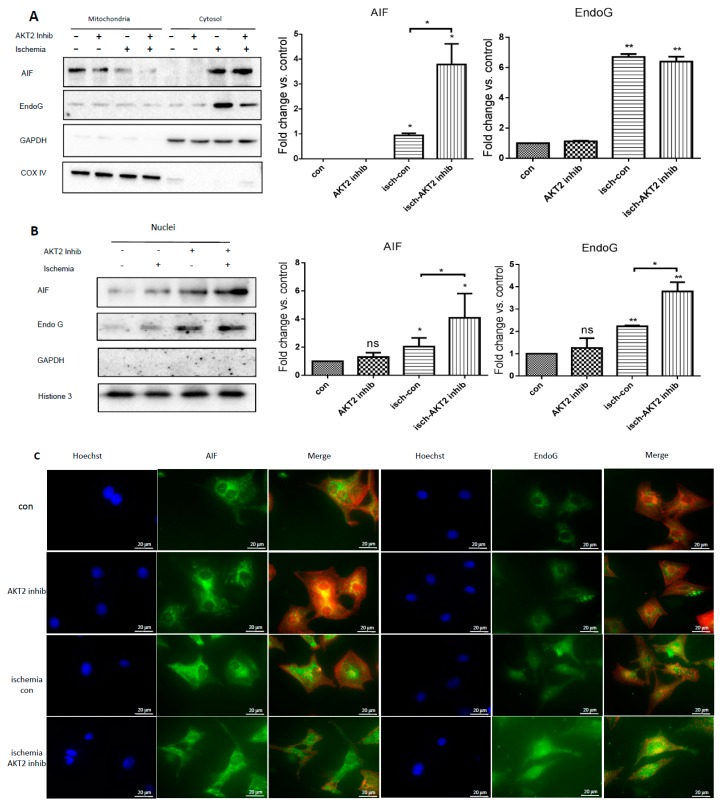
Ischemia induces AIF and EndoG cytosolic and nucleus translocation in cardiomyocytes with AKT2 inhibition. (**A**) left panel: Expression of AIF and EndoG was checked by western blot in cytosolic protein extracts in normal and ischemic cardiomyocytes treated with or without an AKT2 inhibitor; middle and right panel: protein quantification of EndoG and AIF calculated with Image J software; (**B**) left panel: Expression of AIF and EndoG was checked by western blot in nucleus protein extracts in cardiomyocytes with different treatment as in (**A**); middle and right panel: protein quantification of EndoG and AIF calculated with Image J software; (**C**) Immunofluorescence staining of AIF (**green**), EndoG (**green**) and Hoechst (**blue**), α-actinin (**red**), is used as a marker of cardiomyocytes. Scale bar is 20 μm. Each image in this figure is representative of three independent experiments. The bars represent the mean ± SEM of three independent experiments. * *p* < 0.05, ** *p* < 0.01 vs. con; ns: not significant vs. con (one-way ANOVA followed by a Tukey test for post hoc analyses).

EndoG and AIF are two mitochondrial proteins implicated as pro-apoptotic mediators involved in caspase-independent cell death programs by many researchers, and they are proven to be the two main inducers of mitochondrial-related cell death when released from mitochondria and translocated to nucleus [6,11,25,27]. Therefore, the expression of AIF and EndoG was detected in extraction of cardiac cytosolic as well as nucleus protein and also by immunofluorescence staining. Both the cytosolic and nucleus translocations of AIF (Figure 5A–C) and EndoG (Figure 5A–C) were clearly elevated in ischemic cardiomyocytes treated with and without an AKT2 inhibitor, while the increase of AIF and EndoG nucleus translocation was much more significant in AKT2-blocked NRCMs during ischemia (Figure 5B,C). These results suggest that inhibition of AKT2 specifically induces EndoG and AIF nucleus translocation and activates mitochondrial-dependent DNA degradation in ischemic myocardium without caspase activation.

### 2.6. Both EndoG and AIF Are Responsible for Caspase-Independent Cell Death When AKT2 is Blocked

**Figure 6:**
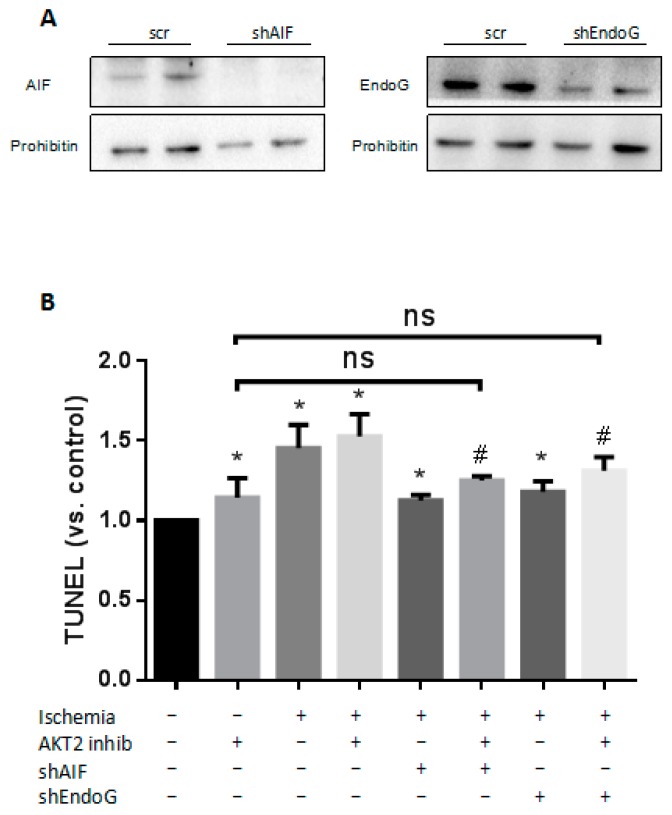
Ischemia induces mitochondrial injury and mitochondrial protein translocation in cardiomyocytes. (**A**) Expression of AIF and EndoG in 40 μg of protein from total extracts of cardiomyocytes transduced, scrambled (scr), and transduced with AIF-specific and EndoG-specific small hairpin RNA interference (shRNAi) construct (shAIF and shEndoG), 5-days post infection. Prohibitin was used as loading control; (**B**) Detection of apoptosis by TUNEL in scrambled-transduced (scr), EndoG-deficient (shEndoG), and AIF-deficient (shAIF) cardiomyocytes treated with or without an AKT2 inhibitor during ischemia. The bars represent the mean ± SEM of three independent experiments. * *p* < 0.05 vs. control, # *p* < 0.05 vs. cells treated with akt2 inhib (one-way ANOVA followed by a Tukey test for post hoc analyses). ns: not significant.

The cell death induced by AIF and EndoG nucleus translocation was defined as a unique process which is distinct from both apoptosis and necrosis due to its specific feature of nucleus DNA degradation [28]. In apoptotic cells, AIF induces chromatin condensation and large-scale DNA damage in a caspase-independent manner when translocating from mitochondria to the nucleus [9,29]. EndoG executes DNA damage triggered by Bcl-2/adenovirus E1B 19 kDa protein-interacting protein 3-like (Bnip3) under cell death stimuli including experimental ischemia [7,11,30]. To ascertain the role of EndoG and AIF in cardiomyocyte apoptosis during ischemia with AKT2 blockage, cardiomyocytes were transduced with lentiviral vectors carrying small hairpin RNA interference (shRNAi) of Endo G and AIF respectively or together. Five days after transduction, cardiomyocytes were exposed to AKT2 inhibitor 3 h prior to ischemia (see “Materials and Methods”). The effectiveness of EndoG and AIF shRNAi vectors were checked by the decrease in AIF and EndoG protein levels measured in protein of cardiomyocyte total cell lysates (Figure 6A). As shown in our data, increased apoptosis was observed in ischemic cardiomyocytes compared with control; suppressed AIF or EndoG expression correlates with a significant reduction of DNA damage to nearly the same level as cardiomyocytes only treated with AKT2 inhibitor during cardiac ischemia (Figure 6B). These results suggest the indispensable role of AIF and EndoG in controlling cardiomyocyte DNA degradation during ischemia when AKT2 is blocked.

## 3. Discussion

This study reveals a novel role for AKT2 in regulating the apoptotic signaling network during myocardium ischemia. Our data demonstrate that AKT2 inhibition induces the silencing of caspase activation which mediates both FADD (Fas-associated death domain)- and mitochondrial-dependent apoptosis during cardiac ischemia. Our data also show that the blockage of AKT2 leads to the stimulation of cell death through promoting nucleus translocation of mitochondrial apoptogenic factors AIF and EndoG in ischemic cardiomyocytes with the absence of caspase activation.

It is well established that the AKT kinase family plays essential roles in protecting the heart from ischemic injury, however, the mechanism of AKT kinase family affording to cardiac protection is complicated due to its diverse array of functions within the cardiovascular system, including apoptosis, proliferation, differentiation, inflammation and autophagy [31,32]. It is proved that AKT2 deficiency in myocardium during ischemia results in increased apoptosis [13,31], but so far no research has investigated the mechanism with respect to the apoptotic signaling pathway. In this study we observed that under normal conditions, there are no morphological changes nor dysfunction of the myocardium with AKT2 absence or blockage in vitro or in vivo. However, during ischemic stimuli, AKT2 plays an important role in cardiomyocyte survival by regulating mitochondrial cell death-related gene expression and translocation.

It is well documented that both necrotic and apoptotic cell death contribute to the pathophysiology of ischemic injury of the myocardium [33]. However, the Anexin V/PI staining studied with flow cytometry analysis in our study suggests that despite AKT2 inhibitor treatment, the majority of cardiomyocytes undergo apoptosis rather than necrosis during ischemia. It is widely accepted that apoptosis can be triggered by a variety of internal or external signals that are deemed as FADD-dependent (extrinsic) and mitochondrial-dependent (intrinsic) apoptosis respectively. In our research, we found that the role of extrinsic apoptosis pathway is inactivated due to the sustained low caspase-8 activity in AKT2-inhibited cardiomyocytes during ischemia. Therefore, we proposed that the intrinsic pathway would be the main mechanism.

Cardiac muscle cells rely on mitochondria to obtain energy and even subtle perturbations in mitochondria content or function can result in cardiac dysfunction [34]. Mitochondria play a key role as apoptosis occurs through the opening of the mitochondrial permeability transition pore that is regulated by Bcl-2 family proteins such as Bax [26,35], and the disruption of mitochondrial outer membrane permeabilization happens secondary to the loss of MOMP during ischemia [8]. The AKT family is an upstream factor of Bax and negatively regulates Bax expression. The function of Bax during ischemia has been well documented, and plays a crucial role in mitochondrial-dependent apoptosis (Bax, cytosolic Cyto C, activated caspase-9 and activated caspase-3) via inducing mitochondrial permeabilization and therefore the release of apoptogenic factors [25,36,37]. As shown in our data, significant increases of Bax protein abundance and disrupted MOMP are in accordance with elevated apoptotic rates observed in cardiomyocytes with AKT2 inhibition during ischemia, suggesting severe disruption of MOMP with additional AKT2 blockage. Furthermore, as it is well-documented that MOMP disruption induces Cyto C intracytosolic translocation which subsequently promotes AIF release from mitochondria [26,38]. Our results show that cytosolic translocation of Cyto C is in parallel with MOMP elevation, which strengthens our conclusion that mitochondrial permeabilization is disrupted by AKT2 inhibition during cardiac ischemia, and the release of mitochondrial Cyto C is a precedent of mitochondrial injury.

We further investigated the role of mitochondrial-dependent (intrinsic) apoptosis in ischemic cardiomyocytes with AKT2 inhibition. The intrinsic apoptosis has been proven to be related to caspase-9 activation upon Cyto C release from mitochondria, and activates proapoptotic caspase-3 thereafter [6]. Of interest, neither activated caspase-9 nor caspase-3 was shown in cardiomyocytes with AKT2 inhibition during ischemia, compared with significantly elevated activity of the two caspases observed in ischemic cardiomyocytes treated without an AKT2 inhibitor. This result suggests that AKT2 blockage is unrelated to the triggering of the intrinsic apoptotic signaling pathway during ischemia.

Although caspases have been widely accepted as important mediators of apoptosis, there is accumulating evidence suggesting the existence of other caspase-independent mechanisms of cardiac cell death upon various stimuli [9]. It is reported that several mitochondria-located proteins related to cell survival are released during ischemic stimuli including AIF and EndoG [11,33,39], which are downstream of Cyto C [26]. Our results reveal that the cytosolic release of AIF and EndoG as well as their nucleus translocation is significantly promoted in ischemic NRCMs with AKT2 inhibition. Furthermore, by using lentivirus-driven short-hairpin knockdown RNA (shRNA) of AIF and EndoG respectively or together in AKT2 inhibitor-treated cardiomoycytes during ischemia, cell death is attenuated and survival is maintained almost the same level as that of cardiomyocytes only treated with an AKT2 inhibitor. These results strengthen our conclusion that, other than cardiomyocytes containing multiple cell death signaling pathways which are both caspase-dependent and -independent during ischemia, there is a switch to the mitochondrial-related DNA degradation that harms myocardium survival during cardiac ischemia when AKT2 is blocked.

The current study is the first that elucidates the function and mechanism of AKT2 in cardiac apoptosis during ischemia. It showed that inhibition of AKT2 activity induces silencing of both the internal and external apoptotic signaling pathway, while upon the disruption of MOMP, mitochondrial-dependent cell death was provoked via conducting AIF and EndoG nucleus translocation and thereafter DNA degradation, thus supporting the conclusion that there is a switch to the unique mitochondrial-dependent DNA degradation pathway during cardiac ischemia when AKT2 is inhibited. These findings illustrate the complex function of AKT2 in the heart and suggest that caution should be exercised when targeting AKT2 as a clinical therapy of myocardium cell death induced by ischemia injury.

## 4. Materials and Methods

### 4.1. Cardiomyocyte Culture, Cardiac Fibroblast Culture and Tissue Samples

The investigation with experimental animals conforms to the Guide for the Care and Use of Laboratory Animals published by the US National Institutes of Health (NIH Publication No. 85–23, revised 1996). The hearts of 4-month-old AKT2 knockout mice and wild type littermates were purchased from the Model Animal Resource Information Platform of Nanjing University (#D000054). We obtained neonatal cardiomyocytes from the hearts of 2–4-day-old Sprague–Dawley rats after digestion with type-2 collagenase (Worthington, Lakewood, NJ, USA). We used a two-round pre-plating in order to deplete cardiomyocyte culture of non-myocardial cells. Cells were plated at a density of 10^3^ cells/mm^2^ in 2 g/L gelatin-coated FALCON polystyrene dishes (Becton Dickinson, Palo Alto, CA, USA) and NUNCLON four-well plates (NUNC, Roskilde, Denmark). The medium used was M199:DMEM (dulbecco’s modified eagle medium) 1:3 (SIGMA-Aldrich, St. Louis, MO, USA), pH 7.2 with 7.2 mmol/L glucose, 10% horse serum and 5% fetal calf serum (Gibco, New York, NY, USA). Cardiomyocyte purity was checked by immunocytochemistry with a cardiac sarcomeric α-actinin monoclonal antibody (clone EA-53, SIGMA-Aldrich, St. Louis, MO, USA), and was found to be higher than 90% after 24–48 h in vitro.

Cardiac fibroblasts were obtained from the first preplating of cardiomyocyte isolation and cultured in DMEM with 10% fetal bovin serum (FBS). Cells from passage 2 and 3 were used for the experiments. Immunocytostaining with antibody to α-actinin identified no positive cells in such cultures. 

Deprivation was initiated after 24 h of plating, by culturing cardiomyocytes in DMEM minimal medium without serum and glucose. We added AKT2 inhibitor CCT128930 (Selleck, Shanghai, China) at different concentrations when indicated. Cardiac fibroblast cells (CFs) was obtained from the first pre-plate of cardiomyocyte isolation. We cultured CFs in DMEM-1 % nonessential aminoacids (SIGMA-Aldrich) and 20% FBS. Different AKT isoform inhibitors were added to the culture medium at the concentrations and times described in the figures.

### 4.2. Experimental Ischemia

Experimental ischemia of NRCMs was performed according to previous protocol which is described elsewhere [3]. Briefly, medium of cultured neonatal rat cardiomyocytes (NRCMs) were changed to Tyrode’s solution (NaCl 137 mM, KCl 2.7 mM, Na_2_HPO_4_ 8 mM, KH_2_PO_4_ 1.5 mM, CaCl_2_ 0.9 mM, and 0.5 mM, initial pH: 7.2) and incubated inside a hypoxic chamber (Billups-Rothenberg) in a mixture of 5% CO_2_ and 95% N_2_ following manufacturer’s instructions to attain a 0.1% oxygen concentration for 12 h.

### 4.3. Histology

Briefly, four mice hearts of each genotype were perfused with paraformaldehyde. Then, the hearts were embedded in paraffin wax. Serial sections were cut to a thickness of 5–7 μm and processed with hematoxylin-eosin staining.

### 4.4. shRNA Knockdown of AKT2, Endo G and AIF

Gene Silencing—In order to obtain the rat-specific small interfering RNA (siRNA) construct, we designed several primer pairs against the target gene; the selected sequences are shown in Table 1. The subcloning strategy is based on choosing a sequence into the target gene, which is 19 nucleotides long. Primer annealing was done in 50 mM Hepes pH 7.4, 100 mM NaCl buffer, using 1 μL of each primer stock (3 mg/mL) to a final volume of 50 μL (primer final concentration 60 μg/m). The protocol for primer annealing consists of progressively reducing the temperature from 90 to 10 °C and increasing time intervals. Annealed primers have protuberant ends compatible with restriction sites for HindIII and BglII (Takara, Dalian, China), ready to be introduced directly into pSUPER.retro.puro (Oligoengine Inc., Seattle, WA, USA) previously digested by HindIII and BglII (Takara). The pLVTHM plasmid is the lentiviral vector used for the transduction of specific shRNAi into mammalian cells. Therefore, the promoter fragment H1 of pLVTHM was replaced by the “H1-shRNA” pSUPER fragment, using the restriction sites for EcoRI and ClaI. Finally, we obtained shRNA constructions into the lentiviral vector and proceeded with the employment of the RNA interference technique to silence the expression of target genes in cardiomyocytes.

We used HEK293T cells as a packaging cell line for lentivirus production [40]. Briefly, the lentiviral siRNA plasmids were co-transfected with packaging system vector psPAX2 and envelope plasmid pM2G in the HEK293T cells. Polyetherimide (SIGMA-Aldrich, 408727) transfection method was used for plasmid co-transfection. The viruses containing siRNA constructs were collected from the supernatant of HEK293T cell culture and were used for transfecting primary cardiomyocytes. The efficiency of lentivirus transfection was supervised by checking GFP with an inverted fluorescence microscope (Carl ZEISS, Axio Vert.A1). The percentage of GFP-positive cells was detected to be between 90% and 99%.

### 4.5. Chemicals

AKT2 inhibitor (Selleck, S2635, 5 μM, 10 μM); pan-caspase inhibitor Z-Val-Ala-Asp (OMe)-CH_2_F (z-VAD-fmk) (Selleck, S7023, 100 μM).

### 4.6. RNA Extraction and Real Time Quantitative RT-PCR

Total RNA was purified with the Trizol Reagent method (Molecular Research Center, Inc., Cincinnati, OH, USA) from 1 to 2 × 10^6^ control cardiomyocytes, following the manufacturer’s instructions. Equal amounts of total RNA were retrotranscribed using ThermoScript reverse transcriptase (Invitrogen, USA) and random hexamers (Roche Applied Science, Rotkreuz, Switzerland). The presence of cFlip and Gapdh (glyceraldehyde-3-phosphate dehydrogenase) transcripts was checked by real-time PCR using specific primers (cFlip Foward 5′–3′: GCAAGGTAGCCAA- GGACAAG; Reverse 5′–3′: TTTGGGAGAGAAGCCTGGAG; Gapdh Foward 5′–3′: AACGACCCCTTCATTGACCT; Reverse 5′–3′: CCCCATTTGATGTTAGCGGG). The annealing temperature was 58 °C, and elongation time was 30 s per cycle, in a GeneAmp PCR System 2700 thermocycler (Applied Biosystems, Foster City, CA, USA).

### 4.7. Western Blot and Immunofluorescence

At the end of the treatments, cells were scraped from the culture dishes, pelleted, and washed with ice-cold phosphate-buffered saline (PBS). After lysis in 95 °C pre-warmed 125 mM Tris, 2% SDS (sodium dodecyl sulfate) (pH 6.8), cell lysates were centrifuged, and the supernatant was used as whole protein cell lysate. Protein concentration was then measured in total and cytosolic extracts by BCA (bicinchoninic acid) assay (Vazyme, E112-02). SDS-PAGE (sodium dodecyl sulfate- Polyacrylamide gel electrophoresis) was performed, and protein was electrotransferred to Immobilon-P filters (Millipore, Bedford, MA, USA, ISEQ00010) and reacted with relevant primary antibodies. Immunoblots were exposed to appropriate peroxidase-conjugated secondary antibodies and developed with the ECL (enhanced chemiluminescence) System (Pierce, #32106) or the SuperSigna Substrate (Tanon, #180-5001).

For immunofluorescence detection, cells were grown in a 4-well plate (NUNC, Roskilde, Denmark) and fixed with 4% paraformaldehyde (PFA), rinsed twice with PBS, and processed as described. Primary antibodies (Table 2) were diluted in blocking solution (bovin serum, 2% horse serum, 0.1% Triton, 2% BSA (albumin from bovine serum) diluted in PBS) and incubated for 2 h at room temperature (RT). Then the wells were washed three times with BS and incubated with the appropriate secondary antibody for 1 h at RT. Finally, cell nuclei were stained with Hoechst 33342 (SIGMA-ALDRICH, #B2883) for 30 min at room temperature. Cells were rinsed twice with PBS and mounted in Vectashield (Vecto Laboratories, Burlingame, CA, USA), which then were observed with inverted fluorescence microscope (Carl ZEISS, Axio Vert.A1, Dublin, CA, USA).

Antibodies—Primary antibodies used in this work are shown in Table 2. For immunofluorescence, secondary antibodies were Alexa Fluor^®^ 594 Goat Anti-Mouse Immunoglobulin G (IgG), Immunoglobulin A (IgA), Immunoglobulin M (IgM) (H+L) (Invitrogen, A11032) and Alexa Fluor^®^ 488 Goat anti-Rabbit IgG (H+L) (Invitrogen, A11008). 

### 4.8. Measuremennt of MOMP

MOMP was monitored using the fluorescent dye Rhodamine 123, a cell permeable cationic dye. Rhodamine 123 was added to cell cultures to attain a final concentration of 10 μM for 30 min at 37 °C at the end of each treatment. The cells were collected and washed twice with PBS and then analyzed by FACScan flow cytometer (BD, FACS Calibur, New York, NY, USA).

### 4.9. Measurement of Apoptosis

After treatment, apoptosis was assessed with FITC (Fluoresceninisothiocyanate) Annexin V/PI kit (BD556547) following the manufacturer’s protocol. Briefly, myocytes were incubated with annexin V-fluorecein isothiocyanate conjugate at room temperature for 15 min in a humid chamber, counterstained with propidium iodide, and observed by flowcytometry (BD, FACS Calibur).

### 4.10. TUNEL Assay

TUNEL (transferase-mediated deoxyuridine triphosphate-biotin nick end labeling) analysis of cultured myocytes was performed with a commercially available kit for detecting end-labeled DNA following the manufacturer’s protocol (Beyotime, #C1086) and observed by flowcytometry (BD, FACS Calibur).

### 4.11. Enzymatic Caspase Activity Assay

Caspases activities were measured using the caspase-8, -9 and -3 activity assay kit (Beyotime, C1115, C1151 and C1157) according to the manufacturer’s instructions. Briefly, cultured cardiomyocytes were washed with cold PBS twice, and resuspended in lysis buffer for 20 min. The lysate was then centrifuged at 16,000× *g* at 4 °C for 15 min. Caspase-8, -9 and -3 activities were measured by using reaction buffer and caspases substrate peptides Ac-DEVD-pNA, Ac-IETD-pNA and Ac-LEHD-pNA respectively. The release of *p*-nitroanilide (pNA) was qualified by determining the absorbance with Tecan SUNRISE (Männedorf, Switzerland) at 405 nm.

### 4.12. Mitochondria, Nuclei and Cytosol Extraction

Cardiomyocytes were washed twice in phosphate-buffered saline (PBS) and once with nuclei isolation buffer (NB) (10 mM Hepes pH 8.0, 10 mM KCl, 1.5 mM MgCl_2_, 1 mM dithiothreitol (DTT) and 1 mM phenyl-methylsulfonyl fluoride (PMSF)). Next, they were suspended in NB, allowed to swell on ice for 15 min and gently lysed with homogenizer. Liberated nuclei were then layered over 30% sucrose in NB and centrifuged at 800× *g* for 10 min, followed by washing in NB and suspension in nucleus membrane lysis buffer (1 M Hepes pH 8.0, 1 M NaCl, 125 mM EDTA (Ethylenediaminetetraacetic acid), 1 mM DTT, 0.2 mM spermine, 1 mM PMSF and 25% glycerol), the lysate was vortexed for 30 s and put on ice for 10 min. Then, the total lysate was centrifuged at 4 °C, 12,000 rpm for 20 min; the supernatant was nucleus lysate which can be used directly or stored at −20 °C for future use.

Mitochondria extraction kit (Beyotime, C3606, Shanghai, China) was used for the isolation of cardiomyocyte mitochondria and cytosol following the manufacturer’s protocol.

### 4.13. Statistics

Data are shown as mean ± SEM (standard error of mean). All statistical analyses were performed using Prism (Graphpad, San Diego, CA, USA) software. Differences between groups were compared by one-way ANOVA followed by a Tukey test for post hoc analyses where appropriate. A *p*-value < 0.05 was considered statistically significant.

## 5. Conclusions

Our results show that AKT2 inhibition induces a unique mitochondrial-dependent DNA degradation pathway by activating nucleus translocation of AIF and EndoG during cardiac ischemia, suggesting a new function and mechanism of AKT2 in regulating cardiomyocyte survival.

## Figures and Tables

**Table 1:** Short-hairpin knockdown RNA (shRNA) primer sequences.

Gene	Oligonucleotides Sequences (5′→3′)
Endo G	gatccccGGAACAACCTTGAGAAGTAttcaagagaTACTTCTCAAGGTTGTTCCttttta
	agcttaaaaaGGAACAACCTTGAGAAGTAtctcttgaaTACTTCTCAAGGTTGTTCCggg
AIF	gatccccGCATGCTTCTATGATATAATTCAAGAGATTATATCATAGAAGCATGCttttta
	agcttaaaaaGCATGCTTCTATGATATAATCTCTTGAATTATATCATAGAAGCATGCggg
Scrambled	gatccccGAATGCTAAGATGTCTAATttcaagagaATTAGACATCTTAGCATTCttttta
	agcttaaaaaGAATGCTAAGATGTCTAATtctcttgaaATTAGACATCTTAGCATTCggg

**Table 2:** Antibodies (Applications used were IF: Immunofluorescence, WB: Western blot).

Antigen	Provider	Catalogue Number	Application
AKT2	Cell Signaling	3063	WB
P-AKT2	Cell Signaling	8599	WB
Bax	Cell Signaling	2772	WB
Cytochrome C	Cell Signaling	4272	WB
AIF	Cell Signaling	5318	WB
Endonuclease G	Abcam	Ab76122	WB
Bcl-2	Wanleibio	WL01556	WB
Cleaved caspase-3	Cell Signaling	9661	WB
Caspase-8	Cell Signaling	9746	WB
COXIV	Cell Signaling	4850	WB
Histon 3	Ruiying Biological	RLM3038	WB
Prohibitin	NeoMarkers	292P501F	WB
GAPDH	BBI Life Sciences	D110016-0200	WB
α-actinin	SIGMA	A7811	IF
